# Ethical perspectives regarding Euthanasia, including in the context of adult psychiatry: a qualitative interview study among healthcare workers in Belgium

**DOI:** 10.1186/s12910-024-01063-7

**Published:** 2024-05-21

**Authors:** Monica Verhofstadt, Loïc Moureau, Koen Pardon, Axel Liégeois

**Affiliations:** 1 End-of-life Care Research Group, Vrije Universiteit Brussel (VUB) & Ghent University, Laarbeeklaan 103, Brussels, 1090 Belgium; 2 Faculty of Theology and Religious Studies, KU Leuven, Louvain, Belgium; 3 Organisation Brothers of Charity, Ghent, Belgium

**Keywords:** End-of-life, Ethics, Euthanasia, Medical assistance in dying, Mental disorders, Values

## Abstract

**Introduction:**

Previous research has explored euthanasia’s ethical dimensions, primarily focusing on general practice and, to a lesser extent, psychiatry, mainly from the viewpoints of physicians and nurses. However, a gap exists in understanding the comprehensive value-based perspectives of other professionals involved in both somatic and psychiatric euthanasia. This paper aims to analyze the interplay among legal, medical, and ethical factors to clarify how foundational values shape the ethical discourse surrounding euthanasia in both somatic and psychiatric contexts. It seeks to explore these dynamics among all healthcare professionals and volunteers in Belgium.

**Methods:**

Semi-structured interviews were conducted with 30 Dutch-speaking healthcare workers who had encountered patients requesting euthanasia for psychiatric conditions, in Belgium, from August 2019 to August 2020. Qualitative thematic analysis was applied to the interview transcripts.

**Findings:**

Participants identified three pivotal values and virtues: religious values, professional values, and fundamental medical values encompassing autonomy, beneficence, and non-maleficence, linked to compassion, quality care, and justice. These values interwove across four tiers: the patient, the patient’s inner circle, the medical realm, and society at large. Irrespective of their euthanasia stance, participants generally displayed a blend of ethical values across these tiers. Their euthanasia perspective was primarily shaped by value interpretation, significance allocation to key components, and tier weighting. Explicit mention of varying ethical values, potentially indicating distinct stances in favor of or against euthanasia, was infrequent.

**Conclusion:**

The study underscores ethical discourse’s central role in navigating euthanasia’s intricate landscape. Fostering inclusive dialogue, bridging diverse values, supports informed decision-making, nurturing justice, and empathy. Tailored end-of-life healthcare in psychiatry is essential, acknowledging all involved actors’ needs. The study calls for interdisciplinary research to comprehensively grasp euthanasia’s multifaceted dimensions, and guiding policy evolution. While contextualized in Belgium, the implications extend to the broader euthanasia discourse, suggesting avenues for further inquiry and cross-cultural exploration.

**Supplementary Information:**

The online version contains supplementary material available at 10.1186/s12910-024-01063-7.

## Introduction

Medical assistance in dying is allowed in 27 jurisdictions in the world and if so, it is mainly restricted to the terminally ill (see BOX 1 in OSF) [1]. Medical assistance in dying entails that a patient’s death request can be granted via *euthanasia*, defined as the intentional termination of life by a physician at the patient’s explicit request, which is currently decriminalised in Australia, Belgium, Canada, Colombia, Luxembourg, the Netherlands, Spain, and New Zealand. In addition, it can be granted by means of *assisted suicide*, also defined as the intentional termination of life by a physician at the patient’s explicit request, but in these cases, the lethal drugs are provided by a physician and self-administered by the patient at a time of the latter’s own choosing (e.g., Australia, Austria, Switzerland, United States). In some countries, not only a physician, but also a nurse practitioner can be involved in the procedure (e.g., Canada, New Zealand).

Euthanasia has been legal in Belgium since 2002, positioning the country as a pioneer in this field with two decades of euthanasia practice [2]. According to Belgian legislation, individuals can be deemed eligible for euthanasia when they are, among other criteria, in a medically futile state characterized by constant and unbearable physical or psychological suffering resulting from a serious and incurable disorder caused by accident or illness [2]. Belgium is one of the few countries that does not exclude people from assisted dying who suffer predominantly from irremediable psychiatric conditions (see BOX 2 in OSF for all legal criteria in Belgium). As regards prevalence, euthanasia accounted for up to 3.1% of all registered deaths in 2023 in Belgium [3]. Whereas most registered euthanasia deaths concerned the terminally ill (approximately 84%), predominantly suffering from cancer, only 48 or 1.4% of euthanasia deaths concerned non-terminally ill adults predominantly suffering from psychiatric conditions. Since euthanasia was legalised, in total 457 such euthanasia cases have been reported, less than 1.5% of all registered euthanasia cases in Belgium [3–9]. 

However, this is only the tip of the iceberg, as there is reason to believe that the total number of requests for euthanasia in Belgium (regardless of outcome), is at least 10 times higher. For instance, recent annual reports from Vonkel, an end-of-life consultation centre in Belgium, revealed around 100 unique patients per year applying for euthanasia for psychiatric reasons. Less than 10% of those euthanasia requests were reported to be carried out [10–12]. Moreover, a recent survey among psychiatrists working in Flanders, Belgium, revealed that 8 out of 10 respondents had been confronted at least once throughout their career with patients requesting euthanasia for psychiatric reasons [13]. The survey also showed that, although three-quarters are supportive of not excluding the option of euthanasia for this specific patient group [14], the majority is hesitant to be actively engaged in a euthanasia procedure [13, 14]. The literature ascribed the reluctance to the complexity of euthanasia assessment in this patient group, inherently high in professional and emotional demands [15–19]. The complexity was for a large part described in terms of the practical considerations surrounding euthanasia requests and assessment, e.g., whether and when these patients can meet the legal criteria.

There is thus reason to believe that healthcare workers’ overarching ethical considerations influence their attitudes on euthanasia in general and in the context of psychiatry specifically, and their practice. As empirical in-depth studies are lacking, this area is largely understudied. To date, only two recent qualitative studies among Dutch physicians emphasised the value-based reasons for euthanasia decision-making, but did not [20] or only summarily [21] scratch the specific context of psychiatry. Another recent qualitative study among Dutch physicians, including psychiatrists, emphasized the value-based reasons for supportive attitudes towards euthanasia, e.g. the value of self-determination, compassion, fairness, and suicide prevention, versus the value-based reasons for not supporting euthanasia, e.g. the mission of medicine of hope and healing [22]. Furthermore, a recent systematic review described the main ethical challenges surrounding the euthanasia practice in the context of psychiatry [23]. However, this ethical debate was mainly concentrated on the permissibility and implementation of euthanasia from a practical-clinical point of view, e.g. whether euthanasia in the context of psychiatry should be permitted, and why the legal requirements can (not) be adequately embedded in the field of psychiatric medicine. How practically and juridically relevant these considerations may be, they remain the outcome of ethical values being weighed up, which means that no single consideration can be considered ethically irrelevant, neutral, or value-free. Moreover, the review was based on articles that have been selected in a timeframe in which sound empirical data regarding euthanasia in the context of psychiatry were largely lacking.

Also, the overarching value-based views of other professionals involved in psychiatric euthanasia practice have not yet been studied. This is striking, as a recent Belgian survey study revealed that that half of the psychiatric nurses (53%) are frequently and directly confronted with such euthanasia requests [24], but in-depth insights into their value-based views are lacking. Furthermore, there are many more formal caregivers, other than psychiatric nurses, involved in euthanasia assessment procedures. End-of-life centres employ e.g., paramedical personnel such as psychologists, psychiatric nurses for intake and registration purposes, and well-trained volunteer personnel such as buddies, entrusted with the task to help these patients to cope with the euthanasia procedure. In addition, rehabilitation-oriented support groups (REAKIRO) were established to help these patients (and their relatives) in walking the tightrope of life and death [25]. All of these caregivers may also have an unacknowledged but influential role in these euthanasia assessment procedures, and therefore, an interesting perspective to reflect on euthanasia legislation and practice. Gaining insight into healthcare workers’ ethical considerations related to euthanasia in psychiatry will lay bare the ethical foundations underlying current practice and is important to inform and spark further debate around this extremely thorny issue, and to promote sound ethical analysis.

Hence, the purpose of this research is to explore healthcare workers’ ethical considerations regarding euthanasia in general and euthanasia concerning adults suffering predominantly from psychiatric conditions in particular.

## Methods

### Theoretical research framework

Our research was guided by the framework of ‘critical social constructionism’ [26], providing a nuanced perspective that diverges from the acknowledgment of an objective reality. This approach intricately examines the interplay of personal, social, and societal dimensions within the phenomena under study. It necessitates an acknowledgment of the layered complexities influencing our understanding of phenomena such as euthanasia, a notion supported by both our prior research [27] and additional studies [23, 28]. 

Our interpretation of the data was informed by social constructionism, which recognizes the role of internalized societal norms in shaping individuals’ perceptions of reality over time. Furthermore, we embraced a contextualist epistemology [29], acknowledging the contextual influence on knowledge formation among both researchers and participants. This methodological approach aimed to capture diverse lived experiences (e.g., diversity in clinical and euthanasia trajectories) and perspectives, including varied attitudes toward euthanasia based on specific relationships (e.g., professional healthcare worker or volunteer). Consequently, we maintained a reflexive stance regarding the potential impact of our individual experiences and identities on our analyses and interpretations, as elaborated in the Ethical Considerations section.

### Study design

The qualitative research design consisted of semi-structured face-to-face interviews with healthcare workers in Flanders and Brussels, Belgium.

### Participants

All participants were Dutch-speaking and had at least one concrete experience with euthanasia requests and procedures concerning adults with psychiatric conditions in the period 2016–2020, either as professional or volunteer healthcare workers. We adopted a broad recruitment approach, with a particular focus on all healthcare providers directly involved in medical practice rather than in managerial or policy-making roles. No further exclusion criteria were employed.

### Recruitment and interview procedure

Purposive sampling was used to ensure diversity and heterogeneity in terms of: participants’ affiliation with institutions holding different stances on ‘euthanasia and psychiatry’; being to a different extent confronted with these euthanasia procedures as regards the amount of experiences (sporadically versus regularly); the nature of the experiences (e.g. confronted with or engaged in euthanasia procedures that were still under review or that had been rejected, granted, performed or withdrawn); and their specific role as professional or volunteer healthcare worker.

Participants were recruited via assistance of our contact persons at: (1) the end-of-life consultation centre Vonkel; (2) the Brothers of Charity; (3) the rehabilitation-oriented centre REAKIRO in Louvain; and (4) the Review Belgian Euthanasia Law for psychological suffering (REBEL) group, a group of Belgian physicians (e.g. psychiatrists), therapists (e.g. psychologists) as well as academics who express their concern on euthanasia in the context of psychiatry via the media. Participants were also recruited via a notice on the sites, newsflashes and/or in the online newsletters of LEIF (Life End Information Forum), Recht op Waardig Sterven (the Flemish Right to Die with Dignity Society) and Vlaamse Vereniging voor Psychiatrie (Flemish Psychiatric Association).

Potential participants contacted MV or a study assistant by phone or mail. The patients were then given an information letter and informed consent form that consisted of 2 main parts. All interviews were conducted by MV or a study assistant, who both have experience in conducting interviews on end-of-life topics. Interviews were held at the participant’s location of choice, except for five interviews which were held online via video call by Whereby^14^ due to the Covid-19 crisis lockdown regulations. Interviews lasted between 55 min and 2 h, and were audio recorded (the online video interviews were recorded by Whereby’s software and immediately transferred in an mp.3 format).

### Measurements

The interview guide (see OSF) contained the following consecutive questions of importance to the present report: (1) What is your personal stance regarding euthanasia as a legalised medical end-of-life option? and (2) What is your personal stance regarding euthanasia in the context of psychiatry?

### Data management and analysis

We used a model of sampling-based saturation, namely inductive thematic saturation, that relates to the emergence of new themes (defined as 7 consecutive interviews without new themes) [30]. We continued to recruit and conduct interviews so that the sample would be heterogenous in terms of socio-demographics, clinical profile, and clinical setting. In particular, our focus was on recruiting individuals with the following profiles: psychologists, male psychiatric nurses and moral consultants/spiritual caregivers employed in residential psychiatric settings (*n* = 5).

All interviews were then transcribed verbatim and de-identified by the interviewers.

We made use of hybrid inductive and deductive coding and theme development by means of a 2-staged process. Stage 1 consisted of an inductive data-driven thematic coding procedure.

We made use of these four phases; (1) identification and coding of all transcripts; (2) the placing of the codes in subthemes, i.e., arguments in favour versus critical concerns; (3) the placing of these subthemes in overarching main themes, i.e., different stakeholders (patient/medicine/society); (4) the comparison and discussion of the findings (with all co-authors). In addition to the inductive approach, we also used a deductive, theory-driven template approach during stage 2. We made use of these four phases; 1) the development of an ethical interpretation framework (see OSF). The framework consists of four key concepts, each involving a multitude of ethical concepts: (a) ethical theories and methodologies, (b) ethical values, (c) basic ethical virtues, and (d) dialogue/decision making ethics; 2) the identification of codes that fit the ethical framework and the theory-driven renaming of these codes; 3) the placing of some of the subthemes in an additional main theme; and 4) the comparison and discussion of the findings (with all co-authors).

#### Ethical considerations

The research team comprised two experienced clinical psychologists, one specializing in euthanasia within the cancer patient population and the other skilled in conducting interviews on this sensitive topic within the adult psychiatric context. Additionally, two ethicists with expertise in assisted dying, including euthanasia, were part of the team. Some authors also have backgrounds in psychiatric practice, including outpatient and residential settings, while others bring expertise through personal experiences. Furthermore, all contributing authors have personal and/or professional connections with individuals navigating death ideation, offering diverse perspectives on euthanasia. Additionally, some authors hold religious beliefs, while others maintain a more agnostic stance. These perspectives vary depending on the predominant viewpoints adopted—whether that of the patient, a close relation, a clinician, an ethicist, or policy stances. To mitigate potential undue influence on data interpretation, three team assemblies were convened. These sessions served to share firsthand encounters from interviews and their outcomes, fostering reflection and deliberation among team members. This proactive measure was implemented to prevent both personal and professional biases from affecting the interpretation of the data.

## Findings

The main characteristics of the 30 participants are listed in Table 1. The sample consisted of 16 physicians, 7 other care professionals (ranging from psychiatric nurses to mobile support teams), and 7 volunteers, all of whom were engaged in one or more euthanasia procedures predominantly based on psychiatric conditions.

**Table 1:** Healthcare workers’ main characteristics (*N* = 30)

Characteristics	Healthcare Workers *N* = 30
Biological Sex	
Male Female	18 12
Age Category	
< 30 years 31-40 years 41-50 years 51-60 years > 61 years	2 2 5 7 14
Type of work environment^1^	
Specialised end-of-life centres Psychiatric units/Psychiatric Hospitals Private or Group Practice Psychiatric Care Homes Other	10 9 5 5 4
Background qualifications^2^	
Psychiatrists General Practitioners (Secretary) consultants at end-of-life centres^3^ Psychiatric nurses Psychologists Moral Consultants/Spiritual Caregivers Buddies Social Workers Experts by experience^4^ Specialist Physicians (other than psychiatrists) Other	10 5 4 3 3 3 3 2 2 2 4
Number of concrete experiences in the year prior to the interview^5^	
1–2 cases 3–5 cases > 5 cases	4 7 19
Physicians’ specific role in recent euthanasia assessment procedures^6^	
Attending/referring physician^7^ Advising physician Performing physician None^8^	7 10 2 1

^1^ Some have more than one work environment

^2^ Some have more than one specific academic and/or professional background or medical end-of-life training

^3^ These people are entrusted with e.g., the patient-intake and referral at end-of-life information or end-of-life consultation centers

^4^ Experts by experience, i.e. people classified with a (proneness to) mental illness, that are trained to provide support for someone who is ‘new’ to the experience or entering rehabilitation approaches

^5^ With concrete experience, it is meant ‘being confronted with and/or actively engaged in a euthanasia procedure, predominantly based on psychiatric conditions’

^6^ Some had experience in more than 1 role

^7^ Some of these physicians hold a normative stance against euthanasia in the context of psychiatry but fulfilled the minimal physician requirement of referring a patient to a colleague-physician upon the patient’s explicit request (*n* = 4)

^8^ This physician expressly refused to fulfill the minimal physician requirement due to conscientious objection

The participating physicians held various roles regarding the handling of euthanasia requests:

- 1 physician refused to discuss the request with the patient on principle grounds.
- 7 physicians managed the clarification of euthanasia requests from their own patients or referred them to colleagues for further clarification.
- 10 physicians provided one of the two legally required formal advices or an additional advice on the euthanasia request.
- 5 physicians performed the act of euthanasia.
- 3 physicians held a more normative, dissuasive stance against euthanasia in the context of psychiatry but were willing to explore and discuss the euthanasia request with the patient.

The sample further included 14 non-physicians, among them members holding one or more roles:

- 2 members were part of mobile teams providing psychiatric care and support in the patient’s home setting.
- 3 were psychiatric nurses working either in a general hospital or in a psychiatric residential setting.
- 2 were Experts by Experience, individuals with a history of mental distress trained to provide support for individuals new to the euthanasia procedure and/or rehabilitation approaches.
- 3 were buddies, individuals entrusted with assisting and supporting the patient throughout the euthanasia procedure.
- 3 were moral consultants/spiritual caregiver, tasked with offering various forms of existential guidance and support to patients considering euthanasia, including religious, moral, and/or other perspectives.
- 5 were consultants at end-of-life information and/or consultation centers responsible for patient intake.

### Participants’ ethical considerations regarding euthanasia, in the broadest context of medicine

As can be seen from the coding structure in Table 2, we ordered coding categories on the level of 1) the individual patient, 2) the patient’s social inner circle, 3) the (para)medical field, and 4) the society. Note that words used verbatim by the interviewees (often interview fragments instead of quotes, as to better illuminate the complexities and nuances of interviewees’ first-hand lived experiences) from the transcribed interviews are incorporated that provide both additional insightful details and reveal the at times interwoven nature of the analysed codes.”

**Table 2:** Healthcare workers’ ethical considerations regarding euthanasia

Stakeholder	Ethical values voiced in favour of euthanasia	Ethical values voiced in critical considerations
The Patient	**Individual Autonomy** - Self-determination - Freedom of choice	**Relativizing Autonomy** - Relational account of autonomy - Bounded rationality due to ‘susceptibility’, ‘subliminality’ - Internalised downside of ‘autonomy’: ‘self-sacrifice’, ‘duty to die’
	**Dignity** - A ‘dignified death’, consistent with one’s own sense of integrity, belief-system, etc. - A ‘good death’: a ‘soft and gentle passing’	**Dignity** - Dignified dying not exclusive to euthanasia
	**Quality of Life/Well-being** - Life not being prolonged unnecessarily - Prevention of meaningless suffering - Continued support to complete (a full and good) life	**Quality of Life/Well-being** - Protection-worthiness of life - Suffering is an inherent feature of human life
	**Compassion** - Relief of meaningless suffering - Cessation of meaningless suffering - Compensation for a life gone wrong	**Meaning and Transformative Value of Suffering** - Providing support in the realisation of self-actualising development amidst the suffering and through hardship/adversity in life - Providing support to find meaning in and acceptance of suffering
The Inner Circle	**Involvement** - Providing support to the patient and her inner circle - Quality of involvement, continued connectedness and care during the euthanasia procedure - Quality for consciously being present and sharing goodbyes at the very end	**Connectedness** - Being aware of the trap of false assumptions: words left unspoken, bottling up own feelings/needs for the sake of the other - Balancing respect for autonomy and individual choice with responsibility and accountability to take care for one another
	**Attentiveness** - Better prepared for the death of a loved one - Better coping and adjusting in bereavement	**Attentiveness** - Continued grappling with unresolved feelings - Protection of the bereaved facing helplessness after a fast-track to death
The field of medicine	**Deontology** - Physician’s duty to provide good (end-of-life) care	**Deontology** ^**2**^ - Physicians’ duty is to save life
	**Responsibility to alleviate suffering** - Evidence of the limits of palliative care as regards to pain relief	**Responsibility to alleviate suffering** - Proper palliative care is available
	**Subsidiarity** - Knowledge on the limits of palliative care	**Subsidiarity** - The use of a palliative filter
	**Professional integrity (executive autonomy)** - Not deciding on life and death, only on allowing and assisting in dying - Pharmacological and technical know-how to end lives	**Professional integrity (executive autonomy)** - Not playing God: not a physician’s mandate to decide on life/death^**2**^ - Pharmacological and technical know-how to save lives
	**Dialogical approach** - Dyadic dialogue (patient-physician)^1^ no intermediary should be tolerated when death is reasonably foreseeable - Triadic dialogue: extended relational autonomy (patient-health professionals-inner circle) when death is not foreseeable	**Medical paternalism**: - Patient’s impaired decision-making capacity and/or undue pressure^**2**^ - Physicians have a more intimate knowledge of the patient and may act in their best interests^**2**^
The society	**Protection** - Legal framework for an ‘underground’ practice before 2002 - Protection against malicious practices - Protection against brutal suicides	**Protection of the most vulnerable** - Especially the mentally ill and the elderly (not an exemption but fully integrated in society) - Extension of scope: first minors then dementia and being tired of life?
	**Dignified dying** - Nascent movement of death literacy and reclaiming control of the dying process - Quality over quantity of life: euthanasia as counterreaction to a prolonged life due to medical advancements	**Dignified dying** - Fast track to death results in the trivialisation of death in the face of ‘Ars Moriendi’ (cf.: fear of dying/death, to avoid suffering, to hold a wake before death) - Romanticised image of euthanasia masks the economics of the death system (‘patients not wanting to be a burden to society’)
	**Solidarity** - Civic engagement preceded the legal framework (points to the need of death literacy)	**Solidarity** - Autonomy as’ societal negligence in disguise’: citizens no longer urged to take care of others (“trek uw plan”) - Equating autonomy and dignity leads to the trap of viewing the ill or the elderly as having ‘undignified’ lives - Neoliberal approach leads to *wealth over health*: the law discourages further investments in health care (‘commodification’ of health care)
		**(distributive) Justice** - Need to uncover existing misperceptions and misconceptions regarding medical end-of-life options

^1^ Only reported by (some) non-physicians

^2^ Only reported by (some) physicians

#### The level of the individual patient

On the level of the individual patient, the following five ethical considerations were distinguished: (1) autonomy, (2) dignity, (3) quality of life, (4) compassion, and (5) the meaning and transformative value of suffering.

First, *Autonomy* was a recurrent theme in all the interviews. Some participants expressly valued *individual autonomy*, and more specifically its following two underpinning characteristics: (1) *self-determination* in terms of the fundamental right for each individual to direct the course of one’s own life, which also includes ‘taking control over the timing and circumstances of one’s end-of-life’, and (2) *freedom of choice*, as they strongly believed that individuals are free to choose what meaning and purpose they assign to their lives. According to them, as each individual should be enabled ‘to live according to one’s own value system’, so should the ending of one’s life also be congruent with one’s own value system. Hence, in their opinion, euthanasia should remain ‘one of the many options to die’.

Other participants called this individualistic approach of autonomy ‘unrealistic’ or even ‘delusional’, as it shies away from: (1) the relational account of autonomy, in which a true autonomous decision was seen as the outcome of a decision-making process which is shaped by individual, social and contextual components, and (2) the internalised downside of autonomy, as the feeling underpinning many euthanasia requests, namely ‘not wanting to be a burden to others’ may lead to ‘self-sacrifice’ and ‘the duty to die’ under the false pretence of autonomy. In addition, some pointed to the power of susceptibility and subliminality, as human beings are subliminal creatures whose behaviour is continuously influenced on both a subconscious and even conscious level. Consequently, internalised pressure cannot be excluded when a patient requests euthanasia. One psychiatrist even stated that ‘*there exists no such thing as a free will, as human beings are always manipulated in many areas of human life and functioning’*.

“I believe that that there should still be places in society where you could die without considering euthanasia. While many people today are facing dementia, and you almost must….

Interviewer: Yes.

“Yes, like how should I deal with it? Should I exit life before it becomes inevitable dementia or something similar? Because I think that in a neo-liberal society, many people internalize the idea that at some point, it becomes a moral duty to step aside. They feel obliged to eliminate themselves. Self-elimination. In a neo-liberal model, as long as you can keep up and contribute, everything is fine. But if you can’t keep up, well, if you cannot fully exercise autonomy, then… Essentially, you should hold your honour and step aside.”

(spiritual caregiver)

Second, participants mentioned euthanasia as an option to *die with dignity*. For those in favour of the Law, euthanasia is considered (1) a ‘dignified way of dying’ when everything that leads up to death, including individual, medical, and social needs and expectations, is consistent with one’s own sense of integrity, belief-system and lifestyle, and (2) a ‘good death’, when referring to the literal meaning of the concept ‘euthanasia’, namely ‘a soft and gentle passing’. Other participants raised concerns on the reference to euthanasia and dignified dying in the same breath, as if “*other ways of dying are not or less dignified*”.

Third, the value of *quality of life* underpinned the arguments made in favour of the Law on Euthanasia, as (1) life itself should not be prolonged unnecessarily, (2) meaningless suffering should be prevented, and (3) a good life should pertain to all stages in life, from the very beginning until the very end, which is feasible if quality of dying circumstances can be guaranteed. As one buddy stated: “*Living a full and good life implies dying a good death*”. Other participants made use of this value underpinning their argument against euthanasia, based on (1) the “protect-worthiness” of life itself and (2) the suffering that must be considered an inherent feature of the human condition.

Fourth, and seamlessly fitting with the former value, divergent courses also emerged regarding the aspect of how to *deal with suffering*. Some participants were in favour of euthanasia out of *compassion* in terms of (1) bringing a kind of relief to the patient when providing her the prospect of an end to the suffering and (2) ending the suffering once it has become ‘useless and meaningless’ and ‘disclosing the limits of the carrying capacity of the self’. Some participants referred to the insufficient degree of quality of life in some patients and valued euthanasia as sort of ‘*compensation for a life gone wrong’.*

Others considered the option of euthanasia as compromising patients’ ability to accept, bear and cope with suffering experiences by offering the opportunity ‘to quickly resign from it’.

Some participants referred to the dynamic features and hence, the potential enriching value of suffering. They believed that one can and must revolt against the perception of pointless suffering, as suffering may offer unique opportunities to achieve personal growth through the realisation of self-actualising tendencies amidst the suffering and though all kinds of hardship and adversity in life. Therefore, the real challenge is to support the sufferer to (re)gain the ability to transform the suffering by means of redefining, accepting, and making sense of it. One psychiatrist referred to the Myth of Sisyphus and stated:

A rock that must be pushed up the mountain, which is terrible, and then Sisyphus lets the rock fall back down, and he must start all over again. And what is the purpose of that suffering? Pushing the rock up? It’s absurd, really, but still. I find it so vital, human, uh, yes. That is something that inspires me enormously and often makes me, well, yes, vitality and suffering, suffering is inherent to being, of course, and one can suffer, of course, that is very serious suffering, terrible suffering. I know that. But well, accept suffering, right? I’m not glorifying suffering, no, I don’t belong to that category. Some Catholics do that; the suffering of Christ, we must… No, not at all. Suffering is inherent to life. Interviewer: It’s just more bearable for some than for others. Interviewee: Then it’s our task to make it more bearable. Yes. (…) Look, that sets a dynamic in motion. By dynamic, I also mean movement. A euthanasia request is often rigid. I am for movement. That’s what Eastern philosophy teaches us too, that everything moves, and we must keep that movement and that the question may change or that people may also discover things. Or indeed, a suffering that is even more exposed, but on which one can then work. There is still much to do, yes, before the ultimate and final act of euthanasia, by a doctor for all sakes, should be considered.

(psychiatrist)

#### The level of the patient’s inner circle

On the level of the social inner circle, the following three ethical considerations were distinguished: (1) involvement, (2) connectedness, and (3) attentiveness.

Some participants stressed that euthanasia can only be a soft and thus ‘good’ way of dying, if the patient’s social inner circle can be *involved* in the euthanasia procedure and if sufficient support to them can be provided. All participants in favour of the legal framework on euthanasia echoed the importance of the social circle being involved in an early stage of the euthanasia procedure, as the prospect of the end of life may challenge a patient’s ability of staying and feeling *connected*. If the euthanasia request is to be carried out, it offers a unique opportunity for both the patient and her social inner circle of consciously being present and sharing goodbyes. Other participants considered this reasoning as potentially deceiving, as concern was raised regarding the trap of false assumptions, in terms of words being left unspoken and the bottling up of one’s own needs for the sake of the other.

As the third doctor, I was asked to provide advice about someone, and the [adult child] was present, a charming [adult child]. The [adult child] was also very friendly but didn’t say much. The man explained why he himself wanted euthanasia and so on. To be honest, at first, I thought, “Well, this won’t take long,” because there were many arguments and reports I had received, but as the conversation went on, I started to feel something different. It turned into a very long conversation, during which the [adult child] also had their say. In short, the father believed that he couldn’t burden his children. He was a kind man who knew what he wanted, and his children were inclined to follow his idea, to follow his vision. However, the children thought, “Yes, we are actually going to agree with our father, and we’ll allow it,” but deep down, they still wanted to take good care of him. The father didn’t want them to take care of him, and there were many other things, but after that long conversation with the [adult child] and the father, and everything else, like, “We’ll still celebrate Christmas together,” there was a complete turnaround. The other physicians involved accepted this very well, and they said, “Okay, for us, it wasn’t clear.

(physician)

In addition, concern was raised regarding the inner circle’s respect of individual patient autonomy and freedom of choice outweighing their r*esponsibility* and *accountabilit*y to take care for one another and to act according to all these subjects’ best interest.

Consequently, divergent discourses on the virtue of *attentiveness* emerged. Whereas for some, the euthanasia procedure may offer a unique opportunity for both the patient and her relatives to be better prepared for death and for the bereaved to better cope with grief, others pointed to the inner circle’s continued grappling with unresolved feelings and perceived helplessness after such a fast-track to death.

Yes, and sometimes I also see people, family members after such euthanasia, yeah, I’ve experienced it several times. They say things like, “Yes, I supported it, but I didn’t know it would affect me like this,” you know? They try to convince themselves, saying, “It was good, it was good, and I stand behind it.” Yeah, you are hardly allowed to do otherwise, but you feel that inner struggle in them, you know? Like, “Was it really okay?” But you can’t question it because you think, “Poor them,” but you still feel it, like, “How sad, how sad.

(psychiatrist)

#### The level of medicine

The following five ethical considerations were distinguished: (1) professional duties, (2) responsibility to alleviate suffering, (3) subsidiarity, (4) professional integrity, and (5) monologic versus dialogic approaches.

First and as regards professional duties, it was (only) reported by some physicians that the physician’s duty is “*to provide good care, which includes good end-of-life care*”. Hence, physicians are the ones who should have euthanasia “as a tool in their end-of-life toolbox”. Others held a different stance and referred to Hippocrates’ Oath when stating that the physician’s duty is to save life at all costs.

Second, all the participants agreed that clinicians have the *responsibility to alleviate the patient’s suffering*. Whereas some welcomed the option of euthanasia due to the experienced limits of palliative care, that in some cases is deemed an insufficient response to intractable suffering, others stated that euthanasia is not needed as physicians have proper palliative care in their toolbox to alleviate all kinds and degrees of suffering.

Third and as regards the *subsidiarity principle*, opinions differed on the use of a palliative filter, i.e., whether a consultation with specialist palliative care units should precede euthanasia.

Fourth and as regards *professional integrity*, some participants relativized the physicians’ executive autonomy. As one psychiatrist stated *“because in the end, we do not decide whether someone might die or not. We only decide whether we want to be of help and assist in it.”* All the ones in favour of the current legal framework echoed that as physicians are the ones that have better access to the lethal drugs and the technical expertise to end the patient’s life in more efficacious ways than non-physicians, they should remain entrusted with euthanasia assessment procedures. Others (only physicians) criticized the Belgian legislator for placing too much power in the physicians’ hands so that the latter “*can play for God instead of using their pharmacological and technical know-how to save lives*”.

Fifth, and as regards the decision-making process, most participants valued the ethical principle of *shared decision-making* between the patient and her physicians, and some even preferred a triadic dialogue in which the patient, her relevant health carers and her social inner circle is involved in euthanasia assessment procedures. For most of them, this type of extended or relational autonomy is considered as best clinical euthanasia practice, especially when death is not foreseeable. According to some non-physicians, a strict dyadic patient-physician approach is to be preferred when death is reasonably foreseeable in a patient with sufficient mental competence. In this event, no intermediary should be tolerated as the medical secret is considered ‘sacred’. One participant elaborated further on this strict dyadic approach and said:

*“*But actually, in my opinion, the request for euthanasia is something between two people. So….

Interviewer: The singular dialogue?

“So, a relationship between the patient and the doctor, yes. That’s what I think. And I do understand that the legislation exists, primarily to protect the doctor against misuse or accusations, because euthanasia used to happen before too, but in secret. But for me as a doctor, it would be enough if a patient whom I’ve known for years, followed for years, maybe 20 years, 30 years, 40 years, and who is terminally ill, asks me in private, ‘I want it.’ For me, it doesn’t need to be more than that for me to say, ‘yes.’ So, there’s no need for a whole set of legislation, except of course to protect myself, maybe from the heirs who might have a different idea about it, yes, but I find it beautiful. And they say, you know, our legislation is such that you can write your euthanasia request on the back of a beer coaster and that’s enough, you know? But how it used to be, euthanasia happened just as well, that’s what I heard from my older colleagues. But it was done in private. Actually, that is the most beautiful sign of trust between a doctor and a patient.*”*

(Physician and consultant)

Others, all physicians without a favourable stance on euthanasia, considered *medical paternalism* morally justified in the end-of-life context, as (1) physicians have more intimate knowledge of the patient and are thus best placed to act in the patient’s best interests, (2) only the independent evaluation from well-trained and experienced physicians may rule out external or internalized pressure from the patient’s social inner circle, and (3) some patients may show impaired decision-making capacity when confronted with the end of life.

#### The level of society

As regards the origins and impact of euthanasia legislation on the level of society, the following four ethical themes emerged: (1) protection, (2) dignified dying, (3) solidarity, and (4) distributive justice.

First and as regards *protection*, some participants valued the existence of a legal framework for an ‘underground’ practice before 2002. According to them, this framework was highly needed to protect the patient against malicious practices and the physician against being charged for murder when ensuring herself that all the legal requirements are met.

So, I believe that it should be well-regulated in a state. In a country, it should be well-regulated. You can either be in favour of it, have reservations, or question it, but when it happens and many people want it or think it’s okay, then it should be regulated. And those, like me, who may be against it, have doubts about it, or wonder, “Is this really necessary?” I would say, or “Does it align with our purpose?” the existential comments that you can make about it, we must accept it because it would be terrible if it, well, it would be even worse if it happened in the underground, like before those laws were established, that’s, yeah. So, I think the laws should exist. Whether I would have made those laws is a different question, or whether I would vote for the parties in parliament that, you know, that support it, that’s another question, but apparently, here in North-western Europe, the need for those practices exists, and it should be regulated properly. And yes, it shouldn’t be left to amateurs or something like that, that’s not the intention. Yes, well, it serves to protect, both in terms of health and to ensure that it doesn’t become a business, of course. I’d prefer it to be integrated into the healthcare system rather than turning it into a profit-driven and exploitative affair for some others. So, that’s….

(psychiatric nurse)

Critical concerns were raised on the lack of protection of the most vulnerable people, i.e., the mentally ill and the elderly. Some of them referred to the amended Law in 2014, that also allowed minors to die by means of euthanasia – be it under more strict circumstances, inter alia, when based on unbearable physical suffering resulting from a medically terminal condition – and feared that the Law will be amended again, so it would no longer exclude the people suffering from dementia or for groups without serious incurable illness, e.g., the elderly with a perceived ‘completed life’.

Second, a major societal shift in thoughts regarding what constitutes *dignified dying* was reported. For some, the Law on Euthanasia reflects a nascent movement of death revivalism, in terms of people reclaiming control over their dying process. In this respect, euthanasia is deemed a counterreaction to the former dominant paternalistic attitude in Western society to systematically marginalise conversations on death and dying, e.g., due to the mechanisms of denial, avoidance, and postponement, and with the line between life and death increasingly held in physician’s hands, which has left many people ill-equipped to deal with dying and death. The current broad public support for euthanasia is seen as the individual patient taking back the decision-making process of dying and death in her own hands. They further considered euthanasia as a logical consequence of living an artificially prolonged life due to e.g., advances in medicine, that have not necessarily enhanced the quality of life.

*“*One thing I also consider is that a part of our lives is artificially prolonged, you know. We don’t live longer because we are healthier, but because we have good pills or better surgical procedures, so we can afford to buy our health. So that part of life is still valuable to me, it’s not less valuable, but it’s artificially extended. So, I think we should keep that in mind, that we can prolong something artificially and maybe even go beyond a point where it no longer works.

Interviewer: Beyond the expiration date?

That’s what I was looking for (laughs). So, in that sense, I believe we should keep in mind that we can artificially extend something and then maybe, even if it’s just that artificial part, stop or be allowed to stop when the person no longer wants to, I think that makes perfect sense.*”*

(psychiatrist)

Others provided arguments against the increased death revivalism, referring to euthanasia as a ‘fast-track to death’ resulting in ‘the trivialisation of death’ in the face of formerly known and experienced Art of Dying. For instance, the current societal tendency to avoid suffering and the fear of dying may lead to patients (too quickly) resigning from a slow track to death, in which there is time to e.g., hold a wake.

But I won’t just grab a syringe, fill it up, and administer a lethal injection, you know? I follow the symptoms. And if they become uncomfortable, then I’ll increase the dosage so they can rest peacefully and not have to suffer. That’s what I call a dignified death. And if the family can be present, sometimes it takes a while for them to arrive, and they’ll say, “Come on, even a dog is not allowed to suffer that long.” Meanwhile, the person is just lying peacefully. But that too. Everything should, even that, should progress, and there isn’t much time left for vigil and, yes, I don’t want to romanticize it, but sometimes you see so much happening between families. There’re all kinds of things happening in those rooms, with the family, reconciliations being made. Memories being shared. “Oh, I didn’t know that about our father.“, an aunt walking in and telling a story. Well, so much still happens. I don’t want to romanticize it, but to say that all that time is useless, that’s not true either. And at the farewell, there’s always, the time, you think there’s time for it, but people are still taken aback when an infusion is given, that it can happen within a minute, even if they’re behind it and have been informed beforehand. Just a minute… and it’s done. The banality of death, it’s almost like that.

(psychiatrist)

These and other participants also criticised ‘the romanticised image of euthanasia’, that masks the economics of the death system, taking financial advantage of ‘patients not wanting to be a burden to society’.

Third and consequently, divergent discourses on the value of *solidarity* emerged. For some, decades of civic engagement pointed to the need of death revivalism and patient empowerment, that resulted in the current legal framework. Others strongly criticised the lack of solidarity underpinning the legal framework on the following three counts: 1) the emphasis on patient autonomy is deemed a ’societal negligence in disguise’, as citizens are no longer urged to take care of others, 2) equating autonomy and dignity in euthanasia debates leads to the trap of viewing the ill or the elderly as having ‘undignified’ lives, and 3) *wealth over health* has become the credo of the current neoliberal society, as the Law on Euthanasia discourages further investments in health care but settles on the ‘commodification’ of health care.

*“*I believe that we should take care of each other and especially care for the most vulnerable in our society. We shouldn’t just leave them to fend for themselves. I don’t think the motto should be all about autonomy, autonomy, and then the flip side, saying, “figure it out on your own.” That’s not acceptable. We have a responsibility to take care of each other. We are meant to care for one another. In biblical terms, we are each other’s keeper, right? “Am I my brother’s keeper?” Yes, I am my brother’s keeper. I must take care of each other, take care of others. So, I think in the long term, speaking maybe 100 years from now, people might say, “Sorry, that was a real mistake in the way they approached things.” I don’t know, but that’s looking at it from a meta-level, as historians call it, “longue durée,” and combining it with a neoliberal model, right? Neoliberalism and euthanasia thinking, it would be interesting to do a doctoral thesis on how they fit together perfectly. How they fit together perfectly… They are no longer patients, they are no longer clients, and I also don’t like the word ‘clients.’ They have become ‘users’. Sorry, but that’s our Dutch translation of the English word ‘consumers’ right? It’s like buying Dash detergent or a car; you buy care, just like the Personal Budget for people with disabilities. You buy your care, sorry, this goes against the very essence of what care fundamentally is. Care is a relationship between people; it’s not something you buy. It’s not something you say, “It’s a contract, and I want that.” It doesn’t work like that. [raising voice] The burden is on society. [end of raising voice] And when the money runs out, you have nothing left. If you can’t buy it, then it doesn’t come. “Here’s your little package,” that’s how it’s translated, and it’s always a hidden cost-cutting operation, let’s be very honest about it, a nice story, but it’s always a hidden cost-saving measure. I see right through that story, but well, big stories are always told, and they are always about saving money. [raising voice] It doesn’t bring anything, right? [end of raising voice] People’s self-reliance, they must stay at home, etc. How many people would benefit from going to a care centre, not at the end of their lives, but just because they feel totally lonely at home, but they can’t get in because nobody wants them there, as they don’t bring any profit.*”*

(spiritual caregiver)

Fourth, critical concerns were expressed concerning the lack of *(distributive) justice* due to the many existing misperceptions and misconceptions regarding medical end-of-life options that need to be uncovered. For instance, many people would be unaware of euthanasia and palliative sedation can both be dignified ways of dying, with euthanasia functioning as a fast-track and palliative sedation functioning as slow track to death. Also, the evolution of death literacy was contested: there was a sense that patients did not become more death literate, as many of them have insufficient knowledge of the content of the many end-of-life documents in circulation.

Yeah, I mean, you see, and I hear many people saying, “My papers are in order.” I won’t say every day, but I hear it almost every day, “My papers are in order.” That’s also something. It’s an illusion of control, right? Because what papers are they talking about? “My papers are in order.” When you ask them about it, they themselves don’t really know what that means, some kind of ‘living will’, ‘an advance care plan’, but yeah, with all… A living will or advance care plan is not that simple either, and then they think, “Oh, if I get dementia and I don’t recognize anyone anymore, they will give me an injection.” Ah yes, but then we are in a different domain, and that’s a whole other… But yeah, people are not well-informed, I find. They have totally wrong ideas and sometimes fear the wrong things, don’t know what is possible and what is not, and they also let themselves believe all kinds of things. Well, there are many misconceptions out there.

(psychiatrist)

### Participants’ ethical considerations regarding the additional procedural criteria for people with a non-terminal illness

As can be seen from the coding structure in Table 3, participants made use of the principle *justice* to motivate their stance on additional (procedural) criteria that people with a non-terminal illness must meet before euthanasia can be carried out, in comparison with people with terminal illness. Those in favour of the additional procedural criteria referred to the differences between the terminally ill and the non-terminally ill regarding the aspect of content (i.e., the difference between general life expectancy and healthy life expectancy) and the aspect of time (i.e., the probability verging on certainty concerning the terminally ill versus the rough estimation concerning the non-terminally ill). Some of them also referred to the legal proceedings and stated that the Law was meant only for people with terminal illnesses to die by means of euthanasia. Others were of the opinion that it concerns only an arbitrary difference due to 1) the vagueness of the concept ‘naturally foreseeable’, i.e., suffering from a terminal illness, and the subjectivity of the calculated course and prognosis of e.g., degenerative somatic illnesses and dementia. A few participants said that this is beside the question, as one’s individual carrying capacity trumps the course and prognosis of an illness.

**Table 3:** Do health care members’ stances differ when euthanasia concerns people with a terminal or a non-terminal illness

	No distinction	In favour of a distinction
The field of medicine	**Justice** - Arbitrary difference due to the vagueness of the concept and the subjectivity of the calculated/expected course of e.g., degenerative illnesses, dementia - Beside the question: one’s bearing capacity trumps the prognosis of an illness	**Justice** - Differences regarding the aspect of time (death is naturally foreseeable or unforeseeable) - Initially, the Law was meant only for people with terminal illnesses

### Participants’ ethical considerations regarding adults with psychiatric conditions

As can be seen from the coding structure in Table 4, when asked about participants’ stances on euthanasia in the context of psychiatry, we distinguished value-based themes at the level of (1) the patient, (2) the field of psychiatry, and (3) society in general.

**Table 4:** Health care workers’ ethical considerations toward euthanasia in the context of adult psychiatry

	Ethical values voiced in favour of euthanasia	Ethical values voiced in critical considerations
The patient	**Justice** - Equality of (end-of-life) care options	**Justice** - Differences in patient profile, e.g., mental competence and the factor of impulsivity, ambiguity, and manipulation
The field of psychiatry	**Justice** - The indissociable unity of psyche and soma) ^**1**^	**Justice** - The inexistence of irremediableness in psychiatry^**1**^ - More caution is needed due to the higher level of ‘subjectivity’
	**Responsiveness to Suffering** - to the unbearableness of ‘invisible’ mental suffering - to the non-alleviability of suffering	**Responsiveness to Suffering** - More time is needed for the therapeutic effect of ‘hope’ to become effective - Differences in the nature and course of somatic versus psychiatric illnesses
	**Protection** - Against brutal suicide - Against ‘therapeutic tenacity’ that often occurs in psychiatry	**Protection** - Preventing suicide conflicts with allowing euthanasia - Against ‘therapeutic negligence’
		**Proportionality** - 24/7 crisis shelter and the therapeutic effect of (yet illegal) drugs should be tried first
The society	**Justice** - The law busts some myths on psychiatric illnesses as ‘Western phenomenon’^2^ - The law busts some myths on the malleability of life and medical omnipotence: society must accept an exit-plan	**(distributive) Justice** - Systemic inequities in mental health care (cf. somatic health care) should be tackled: more budget and resources for accessible and tailor-made mental health care. - Gender disparities
		**Participation** - ‘Social death’: the vicious circle of stigma and self-stigma leading to social exclusion

1 Only reported by (some) physicians

2 Only reported by (some) non-physicians

#### The level of the patient

*Justice* was the main value-based principle that emerged at the level of the patient. Participants in favour of not legally amending additional procedural criteria in the context of psychiatry stated that every patient with a non-terminal illness should receive equal end-of-life care options. The main counterargument given concerned the differences in patient profile, as some questioned whether the mentally ill can meet the legal criteria or stated that extreme caution is needed and thus additional criteria are in place due to the factor of e.g., ambiguity, impulsivity, and manipulation in the mentally ill.

*“I find, the way the procedure is conducted for psychiatric suffering, I find it only natural that they handle it more cautiously because it’s indeed less… It’s not so easy to determine everything, is there really no other option left? And then I understand somewhere that time must be taken to investigate all of that. Because some of these people can be very impulsive, and that impulsivity needs to be addressed somewhere, of course. You also have people who can use their setbacks in the sense of, ‘I’ve been through all that, so I deserve euthanasia.’ And those are the people you need to single out because that’s just… I think those are also people who, with the necessary guidance, can still get out of it. Do you understand? It’s a form of self-pity, in a way. I think there might be resilience there, but they haven’t tapped into it themselves yet; it’s a kind of deflection or something. People with a history of, who say ‘I’ve experienced this and that, so I don’t need it anymore, just give me euthanasia, I deserve that. I’ve been through all that.’ While maybe, if they see, that’s still worth something to me, who knows, maybe that can still happen. They’re people who give up a little too quickly.”*

(Moral consultant)

#### The level of medicine

Regarding the field of medicine, the following four value-based considerations emerged: (1) justice, (2) responsiveness to suffering, (3) protection, and (4) proportionality.

First, and as regards the principle of *justice*, participants in favour of equal procedural criteria for all non-terminally ill pointed to the indissociable unity of soma and psyche. A few physicians went one step further and reported that some psychiatric conditions can be considered terminal, e.g., suicidality, or predominantly of somatic nature, e.g., anorexia. The main counterarguments in this respect were (1) the firm belief in the inexistence of irremediableness in psychiatry (only mentioned by some physicians) or (2) that more caution is needed due to the higher level of subjectivity in terms of diagnostics, prognosis, and outcome.

Second, arguments against the distinction between the somatically versus the mentally ill were based on the attitude of *responsiveness* to the extreme extent and duration of mental suffering that can also render the mentally ill in a medically futile situation and the field of psychiatry empty-handed.

And many of the psychiatric patients I see suffer more than the average ALS patient who has to endure it for three years. In my experience, we’re less advanced in psychiatry compared to most other medical fields. You can easily say “we don’t know” in other areas of medicine and people will understand, but when it comes to psychiatric conditions, it’s different. Doctors might admit “it’s not working” or “there’s no trust,” and they might refer patients elsewhere or even refuse further appointments. I’ve even told a judge during a forced admission, “There’s simply no treatment available.” Yes, sometimes it’s just over and society must accept that there’s no solution. I’m not saying euthanasia is the solution for everyone, but I think it can be an option for some people.

(Psychiatrist)

Other participants were not blind to the deep suffering, but strongly believed in the ground principle and core strength of psychiatry, namely the beneficial effect of hope. In addition, they pointed to the differences in the nature and course of somatic versus psychiatric illnesses when stating that considerably more time is needed in psychiatry, with inclusion of the therapeutic effect of hope to become effective.

*“And I also believe that collectively, within psychiatry, we can and must provide additional support to endure profound despair. So, even in the face of seemingly endless hopelessness, we must maintain hope, look towards the future with trust, and continuously offer encouragement to those who feel hopeless. Our unwavering optimism and support convey the message that together, we can overcome. Because individuals who suffer from severe mental illness are treatable, I consider myself to be a genuinely optimistic psychiatrist. I have witnessed individuals who have harbored feelings of hopelessness and despair for extended periods, sometimes even decades, undergo profound transformations and experience significant improvement, and in some cases, complete recovery.”*

(Psychiatrist)

Third, participants in favour of the current legal framework reported that allowing euthanasia for the mentally ill was needed in the light of *protection*, as it might *protect* the patient against brutal suicides and also against therapeutic tenacity that more often occurs in psychiatry. Other participants in favour of, as well as participants against the current framework held a different stance on the following two counts: (1) allowing euthanasia conflicts with the aim of psychiatry to prevent suicide at all costs, and (2) the mentally ill are insufficiently protected by the Law as there are insufficient built-in safeguards against therapeutic negligence.

But usually with a psychiatric condition, death isn’t imminent. That’s the tricky part, you know? How many suicides do we have here? But anyway, I have an issue with that, using euthanasia as a kind of antidote against, well, against suicide, that’s a completely different matter. But death and psychiatry, why do we have all those government programs against suicide then? Isn’t that dying as a result of a psychiatric condition?

(Psychiatrist, supportive of maintaining euthanasia option in psychiatric settings)

Fourth and as regards *proportionality*, a few participants with a normative stance against euthanasia in the context of psychiatry argued that psychiatric patients may not be allowed to die by means of euthanasia for as long as the field of psychiatry is under-resourced. They pointed to e.g., the lack of sufficient crisis shelters with a 24/7 availability and the lack of palliative approaches in the field of psychiatry. Instead of allowing euthanasia, they argue ‘to jolt the Belgian government’s conscience on mental health policies’. As a revolution to defeat the built-up inequalities in the field of medicine and knowing that palliative and rehabilitation initiatives in psychiatry require time.

*“I oppose euthanasia in psychiatry. Compared to somatic medicine, psychiatry lags behind by 50 years. While physical pain can be managed with medication, there’s insufficient research on treatments for psychological suffering. Promising options like psilocybin and ketamine show potential in easing existential mental struggles. Magnetic stimulation can also alleviate depression, yet access remains limited. Unfortunately, these treatments are underused and under-researched. Many patients aren’t informed about these alternatives to euthanasia. It’s frustrating to see reluctance in exploring these options, especially when they offer hope to long-suffering patients. Utilizing these methods in psychiatric settings carries no risk of addiction. However, current restrictions impede access to these treatments, depriving patients of viable alternatives.”*

(Shortened excerpt from an interview with a psychiatrist)

#### The level of society

When taking a societal perspective, no new arguments emerged from the respondents strongly in favour of the current euthanasia legislation, other than the main value of *justice* described in the subsection above. According to some, the current Law on Euthanasia busts some myths on the malleability of life and medical omnipotence, and even on psychiatric illnesses as a ‘Western phenomenon’, with e.g., depression and suicidality as a consequence of material wealth instead of a neurologic issue in the brain (only reported by some non-physicians).

There are quite a few people who consider the whole issue of the unbearable nature of psychological suffering a luxury problem, you know? They say something like, “Yeah, where are the suicide rates, to put it in equivalent terms, the lowest in the world? In Africa, because they obviously don’t have the luxury to concern themselves with that. They are already happy if they have a potato on their plate every day.” This is a viewpoint held by many, right? They call it a luxury problem, a modern, typical Western luxury problem. And perhaps there is some truth to it, right? But there are other causes of mortality there, which are much higher, such as child mortality, for example.

(non-physician)

Counterarguments were also given and pointed to the value of (distributive) Justice. First, euthanasia was considered as ‘a logical but perverse consequence of systemic societal inequities’ on the one hand and the ‘further evolution towards the commodification or commercialisation of health care in individualised Western societies’ on the other. This would then lead to another vicious circle, with a rapidly growing ‘perception of vulnerable patient groups as irremediable’ and hence less likely to receive potentially beneficial treatment or other interventions. Some took a more radical stance against euthanasia in psychiatry, as they were convinced that euthanasia is nothing but ‘a perverse means to cover societal failures’. In addition, some participants with permissive stances on euthanasia in the context of psychiatry pointed to gender disparities in euthanasia requestors. This was based on the evidence that in the context of psychiatry, many more females request and die by means of euthanasia than males, and proportionally more female patient suffering from psychiatric disorders request and die by means of euthanasia compared to their fellow peers suffering from life-limiting or predominantly somatic conditions.

Finally, some respondents said that they could understand and, in some cases, even support euthanasia in some individual cases, but felt uncomfortable with its impact on the societal level. They pointed to the vicious circle of stigma and self-stigma that may impede the mentally ill to fully participate in societal encounters. In the long run, this type of societal disability may lead to vulnerable patients no longer wanting to perceive themselves a burden to society or to remain ‘socially dead’.

## Discussion

While considering their ethical perspectives towards euthanasia, participants weigh up various values related to and intertwining with the following levels: (1) the patient, (2) the patient’s inner circle, (3) the field of medicine, and (4) society in general. Overall, the participants shared an amalgam of ethical values on each of these four levels, regardless of their stance on euthanasia. It was mainly the interpretation of some values, the emphasis they placed on the key components underpinning each value and the importance they attach to each of the four levels, that determined their stance towards euthanasia. It was uncommon for different ethical values to be explicitly mentioned, which could distinguish distinct stances for or against euthanasia.

As regards euthanasia in the context of psychiatry, the focus has primarily been on arguments for and against euthanasia [23]. However, our study takes a more comprehensive approach, exploring the issue from a wider range of perspectives. This approach allowed us to uncover more complex insights that may have been overlooked if we had only considered it as a black-and-white issue.

Both the systematic review of Nicolini et al. [23] and our study emphasized fundamental ethical domains such as autonomy, professional duties, and the broader implications of euthanasia on mental healthcare. While our findings aligned with those of the systematic review, our inquiry delved deeper into psychiatry-specific considerations, including the influence of sudden impulses and feelings of hopelessness. This underscores the importance of healthcare professionals carefully assessing the timing and contextual aspects of such decisions within psychiatric contexts, ensuring individuals receive timely and tailored support and interventions.

Furthermore, our study extended beyond the boundaries of medical discourse, addressing broader societal ramifications. Participants engaged in discussions about ‘social death,’ a phenomenon that describes the marginalization of individuals despite their physical existence. This discussion highlighted entrenched structural inequities and societal attitudes perpetuating social alienation, particularly affecting marginalized demographics, including individuals grappling with mental health issues. Advocating for societal inclusivity and supportive measures, our study strongly emphasized the need to foster a sense of unity and respect for everyone’s worth, regardless of their circumstances.

### Interpretation of the main findings

We make explicit and discuss the values corresponding to the four classical principles of biomedical ethics, in particular beneficence, non-maleficence, respect for autonomy and justice [31]. We place these values in the context of different ethical approaches, such as religious, professional, emancipatory, social, societal, and virtue-oriented approaches (see the ethical interpretation framework in OSF).

In the discussion section, therefore, the following main values and virtues are addressed: (1) the values of beneficence and non-maleficence in a religious perspective, (2) those same values in the professional context, (3) the value of autonomy in the contemporary emancipation paradigm, (4) the virtue of compassion stemming from virtue ethics theory, (5) the value of quality care in a social approach, and (6) the value of justice in societal policy contexts.

#### Beneficence and non-maleficence: religious perspective

In the realm of euthanasia debates, the interplay of religious beliefs and the values of ‘beneficence’ (the act of doing good) and ‘non-maleficence’ (do no harm) has emerged as a pivotal point of contention, often giving rise to divergent perspectives on this complex ethical issue [32, 33]. Some religious traditions staunchly oppose medical end-of-life decisions, including euthanasia and abortion, viewing them as morally wrong and as disruptive to the natural order of life and death. The principle of ‘sanctity of life’ forms the bedrock of their belief system, underscoring the significance they attach to preserving life at all costs, as an embodiment of beneficence [34, 35]. Conversely, those who argue for the ethical consideration of euthanasia emphasize the concept of beneficence in alleviating suffering and granting autonomy to individuals in their final moments. However, intriguingly, our examination of the topic has revealed a nuanced relationship between religious beliefs and attitudes toward euthanasia. While some individuals in our sample expressed strong religious convictions (*n* = 5) and even considered themselves as practicing Catholics, they did not necessarily adopt a firm normative stance against euthanasia, signifying a complex balancing of beneficence and possible maleficence within their belief system. Conversely, certain participants who held steadfastly against euthanasia (*n* = 3) did not identify with any religious belief system, yet their position was firmly grounded in their perception of potential maleficence associated with medical intervention in life and death decisions. This observation aligns with recent studies highlighting the intricate and multifaceted nature of religiosity, where individuals within various religious frameworks may hold diverse beliefs and values surrounding beneficence and non-maleficence [36, 37]. Moreover, it underscores the powerful influence of societal culture on shaping personal perspectives on euthanasia, and how these views are entwined with the values of beneficence and non-maleficence [36, 37]. 

#### Beneficence and non-maleficence: professional values

Second, a profound division arises between proponents and opponents, particularly in the field of medicine, where interpretations of the Oath of Hippocrates play a central role. At its core, the Oath emphasizes the deontological values of beneficence and non-maleficence, as physicians are bound by a prohibition against administering a deadly drug to ‘anyone,’ even at their explicit request, highlighting the reverence for the sanctity of life inherent in medical practice. This interpretation has led some to perceive active euthanasia as contrary to these sacred principles of preserving life. The notion of beneficence, understood as promoting the well-being of patients, appears to be in tension with the act of intentionally ending a life. Critics argue that euthanasia undermines the fundamental duty of physicians to protect and preserve life. Additionally, the principle of ‘non-maleficence,’ which entails not harming the patient or their life, is seen by some as being in accordance with the ‘sanctity of life’. However, the Oath also recognizes the significance of alleviating relentless suffering, opening the door to a nuanced debate on how these timeless principles align with the modern concept of euthanasia. As the discourse unfolds, perspectives emerge, with some viewing euthanasia as a compassionate form of care, that respects the autonomy and dignity of patients facing terminal illness or unbearable suffering. Advocates argue that euthanasia can be an act of beneficence, providing relief from pain and allowing individuals to die with dignity and control over their own fate. On the other hand, opponents of euthanasia steadfastly uphold the sanctity of life principle, viewing it as an ethical imperative that must not be compromised. They argue that intentionally ending a life, even in the context of relieving suffering, undermines the fundamental values of medical ethics and the intrinsic worth of every human life. For these individuals, euthanasia represents a profound ethical dilemma that conflicts with the near sanctity of medical ethics and the value of preserving life [38–40]. 

### Autonomy: contemporary emancipation paradigm

The principle of autonomy emerges as one of the most prominent and contentious values in our contemporary emancipation paradigm. Autonomy, grounded in the belief in individual self-governance, is often cited as a foundational ethical principle in euthanasia legislation, emphasizing the significance of an individual’s capacity to make choices aligned with their own personal values and desires [31]. However, the discussion on autonomy extends beyond pure individualism, with considerations for relational autonomy, recognizing that individuals are not isolated entities but are shaped by their relationships, communities, and broader societal structures [41]. Within the context of euthanasia, the complexities of autonomy become evident as participants in the debate strived to find a delicate balance. On one hand, they stress the importance of respecting a patient’s individual autonomy in end-of-life decisions, ensuring that their choices are honoured and upheld. Simultaneously, they acknowledge the necessity of accounting for the patient’s social context and broader community when considering euthanasia as a compassionate option. Nevertheless, concerns are raised by some about the potential risks posed by euthanasia legislation, particularly for the most vulnerable individuals, such as the elderly and the mentally ill. These concerns centre on the negative consequences that may arise when individual autonomy is exercised without consideration for others or for societal well-being, and the concept of “social death,” which refers to the marginalization and exclusion of individuals from social relationships and networks due to illness or disability [42, 43]. 

Amidst these complexities, the ethical value of autonomy stands as a paramount consideration. However, its application necessitates thoughtful consideration and balance with other values, including justice, equality, and societal responsibility. Recent reflections on “relational autonomy” have prompted critical evaluations of the idea of pure autonomy, emphasizing the need to delve deeper into the micro, meso, and macro levels that underpin autonomy and address potential conflicts between individual and relational autonomy [44]. Further, it highlights the imperative to take the broader societal context into account when grappling with the ethical challenges associated with euthanasia [45]. 

#### Compassion: virtue ethics

Our study confirms that while the value of autonomy holds importance, it is not the sole determinant in the ethical considerations surrounding euthanasia [46]. In this complex discourse, numerous other ethical values and virtues come to the fore, including the significance of compassion towards suffering individuals and the imperative of alleviating their distress. Notably, compassion is not merely a singular principle, but rather a profound ground attitude or virtue that motivates individuals to empathize with the pain of others and take actions to provide relief.

As revealed in our research, participants who opposed euthanasia did not invoke religious frameworks; instead, they explored diverse philosophical approaches to comprehend suffering and compassion. Among these, non-Western philosophies emphasized embracing suffering as an intrinsic aspect of life, acknowledging the impermanence of all things, including suffering. Additionally, the existentialist perspective of Albert Camus underscored suffering’s innate connection to human existence, leading to deeper self-understanding and comprehension of the world.

These philosophical viewpoints find relevance in the realm of ethics as well. Virtue ethics, in particular, highlights the significance of cultivating virtues such as courage and resilience, while narrative ethics emphasizes storytelling as a means to gain profound insight and reflection on experiences of suffering [47, 48]. Such narratives foster empathy and create a shared sense of experience and community.

Our results show that, for some, suffering may hold positive value in various ways. The nature and intensity of suffering, alongside an individual’s values and virtues, beliefs, and coping capacity, significantly influence the ethics of euthanasia decision-making. An intricate approach that recognizes the multifaceted impacts of suffering becomes essential, acknowledging that various factors could potentially influence the experience of suffering as well as the interpretation of the consequences of the suffering experience. It’s possible that this approach doesn’t solely depend on the quantity of suffering or even its nature. Instead, it could be related to the delicate balance between one’s ability to endure suffering, the burden it places on them, and the (ir)remediableness of this burden, which can vary greatly among individuals as well as it might change over time. Such an approach aims to alleviate relentless suffering and, in certain cases, relieve unnecessary and enduring distress without consistently imposing interpretations upon it. Thus, acknowledging that, experiences of suffering are inherent to life and might act as drivers for personal development, fostering resilience, empathy, and a deeper apprehension of life’s essence, while it also might represent something irremediable, underscores the significance of a broader meaning of the concept of compassion as guiding principle in euthanasia discussions. These discussions further extend to the recognition of the dynamic trajectory inherent to the burden of suffering, as well as its potential for temporal evolution within the individual experiences of the afflicted. Such recognition not only fosters a more intricate understanding of the complex interplay between suffering and resilience but also highlights the acknowledgment that there may be moments when suffering becomes unendurable, surpassing the individual’s capacity to cope. This dimension introduces a layer of intricacy to the ethical considerations inherent in these discussions, thus necessitating a nuanced approach that contemplates the potentialities as well as the constraints of human endurance and the associated ethical ramifications.

#### Quality care: social approach

Examining euthanasia debates from a sociological perspective sheds light on the influence of societal inequalities in healthcare access and quality on the practice of euthanasia, and how it can shape personal, relational, and societal values, leading to the normalization or culturalization of euthanasia [49]. A noteworthy finding in this context is the contrasting perspectives on the evolving process of dying, transitioning from being perceived as in God’s hands to a more medical realm, where proponents of euthanasia view medicine as a catalyst for granting individuals greater control over the timing, manner, and circumstances of their own deaths. They envision the opportunity to be surrounded by loved ones and maintain consciousness while embracing the option of euthanasia, which they believe improves the quality of life at the end.

Proponents also emphasize additional benefits, such as enhanced transparency and regulation, ensuring ethical conduct through regulatory measures. They express concerns about a cultural environment where certain physicians adopt paternalistic attitudes and resist accepting death, prioritizing the extension of life as a moral imperative. In contrast, critical voices argue that death and dying have become increasingly medicalized, leading to their institutionalization. Some critics further contend that this medicalization has devalued the dying process and commodified life itself, leading patients, and families to increasingly rely on medical interventions at life’s end.

Moreover, as shared by some of the interviewees, the growing acceptance of medical assistance in dying may raise concerns. It’s conceivable that this evolving attitude could contribute to a perception of death undergoing a shift in seriousness, resulting in decisions about one’s life conclusion being made with less comprehensive thought and insufficient reflection. Consequently, this scenario could potentially lead individuals who are more susceptible to experiencing feelings of life’s insignificance, weariness, or sense of being ‘through with life’, to lean towards considering euthanasia. However, this inclination might also be driven by a lack of sufficient access to the necessary, long-term quality mental health care that would otherwise facilitate the pursuit of a life imbued with adequate significance, comfort, and dignity, achievable through appropriate (mental) healthcare.

Earlier research indicates that Belgium’s psychiatric care system has been grappling with underfunding and fragmentation, leading to individuals falling through the gaps in the mental health safety net [50]. One critical aspect is, e.g., the inadequate investment in long-term, intensive care, which is precisely the kind of support that individuals grappling with such existential questions may require.

Hence, in the context of euthanasia debates, the value of quality care emerges, encompassing the principle of beneficence, which emphasizes the obligation to provide good care and enhance the overall well-being of individuals. Ethical considerations go beyond the individual’s right to autonomy, extending to societal factors that influence healthcare practices and attitudes towards euthanasia. Addressing the impact of healthcare disparities and the medicalization of dying becomes imperative to ensure ethical and compassionate decision-making that upholds the true value of quality care and respect for human dignity.

#### Justice: societal policy contexts

In the context of euthanasia in somatic versus psychiatric medicine, ethical considerations regarding euthanasia often revolve around the fundamental value of justice [23, 51, 52]. Some respondents in our study emphasized the need for parity between somatic and psychiatric illnesses, recognizing that there should be no distinction between patients suffering from either. They argued that upholding the principle of justice demands equal treatment and recognition of the suffering experienced by individuals with psychiatric illnesses.

However, for others, achieving justice requires acknowledging and addressing the unique challenges faced by patients predominantly suffering from psychiatric illnesses. A comprehensive and integrated healthcare approach is proposed, where mental health is regarded as an integral part of overall health. This approach involves allocating the same level of attention and resources to psychiatric medicine as given to somatic illnesses, aiming to combat stigma and discrimination towards individuals with psychiatric conditions. Equitable treatment during life and at the end of life becomes the focus.

Yet, the Belgian context of psychiatry presents significant challenges. The field is characterized by underfunding and fragmented care, particularly for individuals with longstanding and complex psychiatric problems [53]. Additionally, the end-of-life care for psychiatric patients is still underdeveloped, and palliative psychiatry is in its early stages, lacking a uniformly agreed-upon definition or clear implementation guidelines [54]. In response, Belgium is exploring the “Oyster Care” model, designed to provide flexible, personalized care for individuals with severe and persistent mental illness who may be at risk of neglect or overburdened by psychiatric services [55]. This model aims to create a safe “exoskeleton” or supportive environment for patients, recognizing that recovery, reintegration, and resocialization might not be attainable for everyone with certain psychiatric conditions [55]. 

However, the integration of Oyster Care in today’s psychiatric practice is still limited and requires further development. Emphasizing the value of justice calls for continued efforts to enhance and refine psychiatric care, ensuring that individuals with psychiatric illnesses receive equitable treatment throughout their lives, including end-of-life care decisions [55, 56].

### Implications for future research, policy, and practice

In terms of policy and practice, our findings indicate that the discourse surrounding euthanasia extends beyond legal or medical considerations and encompasses fundamental ethical values that underpin our society. These values may not always be aligned and can create ethical dilemmas that are challenging to address. A value-centred approach to the euthanasia debate necessitates a constructive ethical dialogue among various actors involved, including patients, healthcare practitioners, and the wider community. This conversation should strive to comprehend the diverse values involved and endeavour to achieve a balance between these values. Additionally, ethical dialogue might encourage individuals to reflect on their own assumptions and beliefs, leading to more informed and thoughtful decision-making on ethical and moral issues. Ultimately, ethical dialogue can promote a more just and equitable society that prioritizes empathy, understanding, and mutual respect.

It is also crucial to acknowledge that patients with somatic illnesses and those with psychiatric illnesses may have different needs and expectations regarding the end of life. Hence, end-of life healthcare must be sensitive to the unique needs of each group. This recognition of differences does not justify unequal treatment or discrimination based on the type of illness. Instead, it involves addressing the different needs and expectations of each patient group while ensuring equitable and high-quality care for all.

As regards research, most articles on euthanasia legislation to date placed the emphasis on what other countries and states can learn from the Belgian and Dutch euthanasia practice. In addition, what can be learned is mainly restricted to the evidence and reflections on factual issues from a global practical-clinical perspective. Consequently, one of the main ethical, clinical, and societal issues remains unrequited, namely the impact of legislation and its consequences on an intrapersonal, interpersonal, medical, social, and societal level. Although cultural diversity is recently put high on the research agenda concerning general health care and mental health care, it is largely understudied in the context of end-of-life decisions and largely ignored in the context of psychiatry. Fewer articles have focused on what the latter countries may learn from those not implementing or not considering euthanasia legislation. In an increasingly diverse society, rapidly evolving in terms of fluidity and multi-ethnicity, cross-cultural research can help us learn from one another. To address the many dimensions of euthanasia, there is a need for input from a variety of academic fields, including sociology, anthropology, communication studies, and history. Further interdisciplinary research in all these areas could help inform policy and practice related to euthanasia.

### Strengths and limitations

This is the first empirical in-depth interview study that uncovered the underlying ethical considerations of a variety and relatively large sample of health care professionals and volunteers in Belgium, a country with one of the most permissive legislative frameworks regarding euthanasia, as – unlike in some other countries – it does not exclude adults with psychiatric conditions per definition. Belgium is also one of the pioneering countries with such a legislative framework and can boast on two decades of euthanasia legislation and implementation.

We succeeded in providing a unique and representative sample of participants, varying in gender, work setting and expertise, and stances regarding euthanasia. Finally, and unlike former scientific studies that focused on either the somatic or psychiatric context, we now gauged for participants’ ethical perspectives on euthanasia in both fields of medicine.

There are also several limitations to our study. We may have experienced selection bias, as our sample of non-physicians had varying ages, but the sample of physicians was mostly older than 60. In addition, some interviews had to be postponed or cancelled due to COVID-19 restrictions and, potentially, due to legal and emotional concerns surrounding a high-profile euthanasia case being brought to court. Additionally, our sample exhibited heterogeneity regarding worldview (religious or non-religious), but possibly not regarding other culture-sensitive aspects, like migration background. As our qualitative research focused on exploring themes, narratives, and shared experiences rather than on ensuring high participation rates for statistical generalizability, drawing definitive conclusions regarding the prevalence of each opinion (pro/ambivalent/critical/against), the level of experience, or perspective across the entire spectrum of euthanasia practice is beyond the scope of our study.

Finally, although there is a growing number of countries and states around the globe with a legislative framework on euthanasia, all the legal frameworks differ from one another, so the results of our study cannot be generalized to the specific euthanasia context in e.g., Switzerland or Canada.

## Conclusion

Our study illuminates the foundational values guiding perceptions of euthanasia, including autonomy, compassion, quality care, and justice, which permeate through four interconnected tiers: the patient, their inner circle, the medical community, and society at large. Despite varied stances on euthanasia, participants demonstrated a convergence of ethical principles across these tiers, shaped by nuanced interpretations and considerations. While explicit discussions of distinct ethical values were infrequent, their profound impact on euthanasia perspectives underscores the importance of ethical discourse in navigating this complex issue. By fostering inclusive dialogue and reconciling diverse values, we can promote informed decision-making, justice, and empathy in end-of-life care, particularly in psychiatric settings. Interdisciplinary research is essential for a comprehensive understanding of euthanasia’s dimensions and to inform policy development. While our study is rooted in Belgium, its implications extend to the broader euthanasia discourse, suggesting avenues for further exploration and cross-cultural understanding.

### Electronic supplementary material

Below is the link to the electronic supplementary material.

Supplementary Material 1

## Data Availability

The datasets generated and/or analysed during the current study are not publicly available due reasons of privacy and anonymity, but are available from the corresponding author on reasonable request, following procedures from all 3 Medical Ethics Committees involved. To access the supplementary materials, see the Open Science Framework repository at https://osf.io/26gez/?view_only=af42caddb2554acfb7d1d5aabd4dec7a. Upon publication of this paper, the repository will be made public, and a shorter link will be provided.

## Declarations

### Transparency declaration

MV is the guarantor of the manuscript and affirm that the manuscript is an honest, accurate, and transparent account of the study being reported; that no important aspects of the study have been omitted; and that any discrepancy from the study have been noted and explained in the manuscript.
