# Supplementary material for: Ethical perspectives regarding Euthanasia, including in the context of adult psychiatry: a qualitative interview study among healthcare workers in Belgium
Source: BMC Med Ethics. 2024 May 21;25:60. doi: 10.1186/s12910-024-01063-7 (PMC11107029; doi:10.1186/s12910-024-01063-7)
Supplement: Supplementary file 1 — Supplementary Material 1 [file 12910_2024_1063_MOESM1_ESM.doc]

**Topic list mental healthcarers**

**1. What is your personal stance regarding euthanasia as a legalised medical end-of-life option in the context of somatic medicine?**

**Prompt: How do you feel about the legislature making a procedural distinction between the terminally ill versus the non-terminally ill?**

**2. What is your personal stance regarding euthanasia in the context of psychiatry?**
